# Predictors of left ventricular ejection fraction recovery after guideline-directed medical therapy in patients with newly diagnosed dilated cardiomyopathy and baseline LVEF ≤35%

**DOI:** 10.3389/fcvm.2026.1767079

**Published:** 2026-06-12

**Authors:** Yang Pu, Yaowu Liu, Ningyue Zhang, Yuanqi Yang, Yingming Zhao, Didi Zhu, Xudong Li, Yunfeng Song, Jinling Fu, Shaojie Shen, Long Chen

**Affiliations:** 1 Cardiovascular Department, Zhongda Hospital, School of Medicine, Southeast University, Nanjing, Jiangsu, China; 2 Cardiovascular Department, Zhongda Hospital, Southeast University, Nanjing, Jiangsu, China; 3 Nanjing University Medical School Affiliated Nanjing Drum Tower Hospital, Nanjing, Jiangsu, China

**Keywords:** apolipoprotein A1, dilated cardiomyopathy, ejection fraction, guideline-directed medical therapy, implantable cardioverter-defibrillator

## Abstract

**Background:**

Patients with newly diagnosed dilated cardiomyopathy (DCM) and a baseline left ventricular ejection fraction (LVEF) ≤35% generally have a poor prognosis. Early identification of patients who are unlikely to recover LVEF after guideline-directed medical therapy (GDMT) is clinically important for risk stratification, closer follow-up, repeated LVEF assessment, and timely reassessment of guideline-based device therapy eligibility.

**Methods:**

This retrospective cohort study enrolled 198 patients with newly diagnosed dilated cardiomyopathy (DCM) and baseline left ventricular ejection fraction (LVEF) ≤35%, all of whom received standardized guideline-directed medical therapy (GDMT) for 3∼6 months. Baseline and follow-up echocardiographic, electrocardiographic, clinical, and laboratory data were collected. The primary outcome was recovery of LVEF to >35% after treatment. The secondary outcome was the first occurrence of cardiovascular death or rehospitalization for heart failure during follow-up. Multivariable Firth Logistic regression was used to identify independent predictors and to develop a prediction model, which was internally validated using the area under the receiver operating characteristic curve (ROC AUC), the Hosmer–Lemeshow goodness-of-fit test, and decision curve analysis. Cox proportional hazards regression was used to evaluate predictors of long-term prognosis, and time-dependent ROC analysis was performed.

**Results:**

Multivariable Firth logistic regression showed that absence of left bundle branch block (LBBB) and higher apolipoprotein A1 (ApoA1) levels were independently associated with LVEF recovery to >35%. The presence of LBBB was strongly associated with a lower likelihood of LVEF recovery (OR = 0.02, 95% CI 0.00–0.13, *P* < 0.001), whereas higher ApoA1 levels were associated with a greater likelihood of recovery (OR = 1.40 per 10-unit increase, 95% CI 1.06–1.86, *P* = 0.019). A model integrating LBBB, ApoA1, and age demonstrated good discrimination, with ROC AUCs of 0.93 in the training set and 0.88 in the validation set, good calibration, and positive net benefit on decision curve analysis.

**Conclusions:**

The model may help with early risk stratification, closer surveillance, repeated LVEF assessment, and timely reassessment of guideline-based ICD eligibility. However, external validation is required before routine clinical application.

## Background

Dilated cardiomyopathy (DCM) is characterized by left ventricular or biventricular dilation with systolic dysfunction not explained by abnormal loading conditions or significant coronary artery disease, and clinically manifests with progressive cardiac enlargement, impaired systolic function, heart failure, arrhythmias and conduction disturbances, thromboembolic events, and sudden cardiac death. Its etiology includes primary (genetic) and secondary (acquired) causes such as viral myocarditis, toxic exposures, endocrine disorders, peripartum cardiomyopathy, and immune-mediated diseases. Despite advances in pharmacotherapy that have improved prognosis and quality of life, DCM remains an important contributor to cardiovascular morbidity, mortality, and sudden cardiac death (1, 2).

For patients with DCM and heart failure with reduced ejection fraction (HFrEF), the 2023 ESC guidelines recommend five foundational pharmacotherapies—angiotensin receptor–neprilysin inhibitors (ARNIs) or ACE inhibitors, β-blockers, mineralocorticoid receptor antagonists, and SGLT2 inhibitors—which reduce mortality, symptoms, and the risks of hospitalization and sudden cardiac death. Implantable cardioverter-defibrillators (ICDs) remain one of the most effective strategies to prevent sudden cardiac death (3, 4).

Left ventricular ejection fraction (LVEF) is a key prognostic marker in DCM, and although LVEF ≤35% commonly indicates consideration for ICD implantation, a subset of patients experience meaningful LVEF recovery after guideline-directed medical therapy (GDMT). Consequently, ICD implantation is generally deferred until patients have received optimized GDMT for 3–6 months, after which persistent LVEF ≤35% and NYHA class II–III symptoms guide device decisions. Early identification of patients unlikely to recover LVEF >35% after GDMT may help clinicians recognize individuals at high risk of persistent LV systolic dysfunction, strengthen follow-up, repeat LVEF assessment, and reassess guideline-based device therapy eligibility in a timely manner. However, predictors of LVEF improvement remain incompletely characterized, and prior studies have suggested associations with baseline LVEF, ventricular dilation, LBBB, and biomarker profiles (4–12).

This study aims to identify predictors of LVEF recovery to >35% among hospitalized, newly diagnosed DCM patients with baseline LVEF ≤35% who receive GDMT. We also evaluated longitudinal LVEF changes and clinical outcomes during follow-up to explore the prognostic relevance of these predictors.

## Methods

Data are available from the corresponding author on reasonable request. We conducted a retrospective observational cohort including all consecutive patients hospitalized for heart failure and diagnosed with DCM at Zhongda Hospital, Southeast University, and Nanjing Drum Tower Hospital between January 2015 and April 2024. The study was approved by the institutional ethics committees with a waiver of written informed consent. All procedures adhered to the Declaration of Helsinki.

### Study population

This study was a retrospective observational analysis conducted between January 2015 and April 2024 at Zhongda Hospital affiliated with Southeast University and Drum Tower Hospital affiliated with Nanjing University Medical School.

The inclusion criteria were as follows: (1) patients aged ≥18 years; (2) patients diagnosed with DCM who were hospitalized for heart failure for the first time; (3) baseline and follow-up echocardiographic evaluations performed using the Simpson or biplane method for LVEF, with baseline LVEF ≤35%; and (4) continuous and regular oral administration of guideline-directed heart failure medications for at least 3–6 months after discharge.

**Figure 1:**
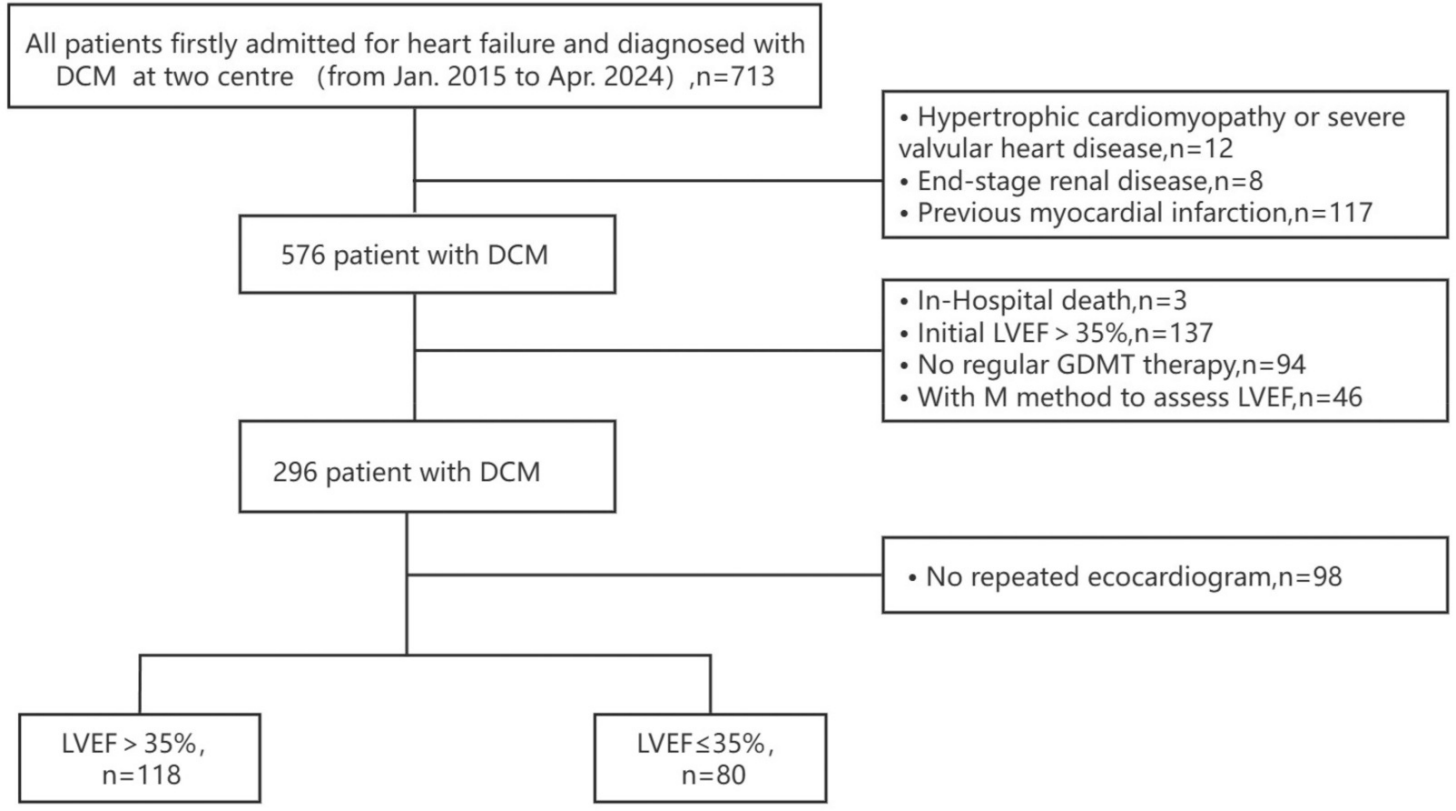
Flowchart of study design. DCM indicated dilated cardiomyopathy; LVEF, left ventricular ejection fraction; GDMT, guideline-directed medical therapy.

Exclusion criteria included: (1) severe ischemic heart disease (≥75% stenosis in the left main or left anterior descending artery, or a history of at least one myocardial infarction); significant cardiovascular volume overload (e.g., uncontrolled hypertension or severe primary valvular heart disease); (2) LVEF assessed using methods other than the Simpson or biplane method; (3) implantation of any cardiac device (including cardiac contractility modulation devices, pacemakers, implantable cardioverter-defibrillators, or cardiac resynchronization therapy defibrillators); (4) comorbidities such as severe anemia, hyperthyroidism, or malignancy; (5) missing more than 30% of baseline clinical data that could not be retrieved; and (6) inability to establish follow-up contact. Figure 1 shows the flowchart of study design.

### Data collection

Clinical data were retrospectively collected from electronic medical records at two centers using standardized forms. Baseline variables included age, sex, BMI, smoking and alcohol use, and comorbidities such as diabetes, hypertension, CAD, prior stroke, AF, and COPD. Fasting peripheral blood samples (≥8 h) were analyzed for hematologic and biochemical parameters: complete blood count (e.g., WBC, RBC, hemoglobin, platelets), renal function (creatinine, uric acid), glucose metabolism (fasting glucose, HbA1c), lipid profile (total cholesterol, triglycerides, LDL-C, HDL-C), liver enzymes (ALT, AST), and cardiac biomarkers (NT-proBNP, D-dimer).

Echocardiographic assessments included left atrial (LA) diameter, left ventricular end-diastolic diameter (LVEDD), end-systolic diameter (LVESD), and LVEF, calculated using the biplane Simpson method from apical four- and two-chamber views. All echocardiograms were reviewed by an experienced cardiologist.

Electrocardiographic data included heart rate, QRS duration, QT interval, bundle branch block, and conduction abnormalities. Discharge medications were recorded, including diuretics, MRAs, ACEIs, ARBs, ARNIs, β-blockers, and SGLT2 inhibitors.

Follow-up data were obtained via telephone interviews and included LVEF at 3–6 months after discharge, subsequent LVEF trends, cardiovascular death, rehospitalization for heart failure, and ICD implantation, which was recorded descriptively.

### Studying endpoints and definition

The primary outcome was recovery of LVEF to >35% after 3–6 months of GDMT. The secondary outcome was the first occurrence of cardiovascular death or rehospitalization for heart failure during follow-up.

### Statistical analysis

Categorical variables are reported as number (%) and compared with the *χ*^2^ or Fisher exact test, as appropriate. Ordinal variables were analyzed with the Mann–Whitney *U*-test. Continuous data are expressed as mean ± SD when normally distributed or median (IQR) otherwise, and compared with the Student *t*-test or Mann–Whitney *U*-test.

Independent determinants of left-ventricular systolic function improvement were identified by multivariable firth logistic regression. Variables with *P* < 0.05 in univariable analysis, together with prespecified clinical covariates (age, sex, baseline LVEF, BMI), entered a stepwise model based on the Akaike information criterion. For internal validation, the dataset was randomly partitioned into training and validation sets in a 7: 3 ratio with a fixed random seed.

Model discrimination was quantified by the area under the receiver-operating-characteristic curve, and calibration by calibration plots and the Hosmer–Lemeshow test. Clinical usefulness was assessed with decision-curve analysis.

Time-to-event outcomes were examined with Cox proportional-hazards models; the proportional-hazards assumption was verified by Schoenfeld residuals. Kaplan–Meier curves with log-rank tests illustrated survival differences for significant predictors. Time-dependent ROC curves and bootstrap-corrected calibration plots (1, 3, and 5 years) further evaluated prognostic accuracy.

All analyses were conducted in R 4.3.0 (R Foundation for Statistical Computing, Vienna, Austria). Full details of packages and functions are provided in Supplementary Table S1.

## Result

### Characteristics

Tables 1, 2 summarize the baseline characteristics of patients grouped by whether their left ventricular ejection fraction (LVEF) recovered to >35% after medical therapy. A total of 198 patients were included, with 146 males and 52 females. Of these, 118 achieved LVEF recovery (>35%), while 80 did not. The median age of the cohort was 55 years (IQR: 41–68.75). Patients in the non-recovered group were significantly older than those in the recovered group [62 years (IQR: 53.5–71.5) vs. 49 years (IQR: 37–60), *P* < 0.001]. No significant differences were found between groups regarding BMI, smoking or alcohol use, hypertension, diabetes, prior stroke, or COPD (*P* > 0.05).

**Table 1:** Baseline characteristics, laboratory test, medication, and imaging data.

Variables	Non-recovered	Recovered	*P*-value
Number of patients	80	118	
Gender, *n* (%)			0.325
Male	56 (70.00)	90 (76.27)	
Female	24 (30.00)	28 (23.73)	
Age,y	62.00 (53.50, 71.50)	49.00 (37.00, 60.00)	**<**.**001**
BMI, kg/m^2^	24.41 (22.63, 28.04)	26.24 (22.68, 29.31)	0.163
Smoking, *n* (%)	28 (35.00)	52 (44.07)	0.202
Drinking, *n* (%)	24 (30.00)	33 (27.97)	0.756
Hypertension, *n* (%)	14 (17.50)	11 (9.32)	0.783
Diabetese, *n* (%)	19 (23.75)	20 (16.95)	0.238
Stroke, *n* (%)	5 (6.25)	7 (5.93)	1.000
Atrial Fibrillation, *n* (%)	5 (6.25)	10 (8.47)	0.562
COPD, *n* (%)	4 (5.00)	2 (1.69)	0.363
Laboratory Test			
TG, mmol/L	1.08 (0.82, 1.37)	1.21 (0.97, 1.77)	**0**.**017**
TC, mmol/L	3.93 ± 1.01	4.13 ± 1.06	0.195
HDL-C, mmol/L	1.04 (0.81, 1.19)	0.97 (0.76, 1.21)	0.318
LDL-C, mmol/L	2.44 ± 0.86	2.54 ± 0.82	0.385
ApoA1, 10 g/L	9.80 (8.18, 11.40)	10.40 (8.65, 12.10)	0.087
ApoB, g/L	0.80 ± 0.23	0.88 ± 0.28	**0**.**035**
Lp(a), mg/L	138.50 (67.00, 266.00)	148.50 (64.25, 339.25)	0.667
RC, mmol/L	0.47 (0.31, 0.57)	0.56 (0.34, 0.86)	**0**.**037**
Albumin, g/L	37.58 ± 4.14	38.70 ± 4.24	0.067
Serum potassium, mmol/L	3.80 (3.61, 4.06)	4.04 (3.74, 4.36)	**0**.**001**
Creatinine, μmol/L	83.50 (70.75, 99.00)	84.00 (68.50, 106.00)	0.606
Glu, mmol/L	5.49 (4.82, 6.54)	5.10 (4.60, 5.92)	**0**.**036**
D-Dimer, μg/L	439.00 (255.25, 880.25)	453.50 (229.25, 1,070.50)	0.716
NT-proBNP, pg/mL	2,090.00 (853.75, 4,800.00)	999.00 (594.00, 2,250.00)	**0**.**001**
Anti-HF Drug Therapy			
β-Blocker, *n* (%)	74 (92.50)	114 (96.61)	0.334
SGLT-2i, *n* (%)	40 (50.00)	86 (72.88)	**0**.**001**
ACEI/ARB, *n* (%)	76 (95.00)	114 (96.61)	0.844
Diuretics, *n* (%)	79 (98.75)	115 (97.46)	0.905
Aldosterone receptor antagonist, *n* (%)	80 (100.00)	117 (99.15)	1.000
ARNI, *n* (%)	76 (95.00)	114 (96.61)	0.844

ApoA1 values were scaled by a factor of 10 (i.e., ORs per 10 g/L).

*P*-values indicate comparisons between patients with and without LVEF recovery after 3–6 months of GDMT. ApoA1 values were scaled by a factor of 10 for regression analyses. ACEI indicates angiotensin-converting enzyme inhibitor; ARB, angiotensin II receptor blocker; ARNI, angiotensin receptor–neprolysin inhibitor; SGLT-2i, sodium-glucose cotransporter-2 inhibitors; TG, triglyceride; TC, total cholesterol; HDL, high-density lipoprotein; LDL, low-density lipoprotein; ApoA1, apolipoprotein A1; ApoB, apolipoprotein B; Lp(a), lipoprotein(a). The bold values indicate that *P* is less than 0.05, which is considered statistically significant.

**Table 2:** Baseline of imaging data.

Variables	Non-recovered	Recovered	*P*-value
Number of patients	80	118	
Eco-cardiography			
LVEF, %	25.75 (21.00, 30.00)	28.00 (25.00, 31.00)	**0**.**008**
RV, cm	2.75 (2.39, 3.16)	2.84 (2.50, 3.77)	**0**.**046**
LV, cm	6.81 ± 0.79	6.63 ± 0.70	0.079
LVESD, cm	6.04 ± 0.96	5.83 ± 0.72	0.097
LVEDD, cm	7.02 ± 0.87	6.72 ± 0.71	**0**.**010**
Electrocardiogram			
Heart-rate	88.79 ± 18.01	89.83 ± 16.84	0.678
QRS time, ms	136.00 (117.50, 160.00)	102.00 (92.00, 110.00)	**<**.**001**
QTc time, ms	479.00 (458.75, 511.25)	447.00 (422.00, 475.00)	**<**.**001**
LBBB, *n* (%)	58 (72.50)	3 (2.54)	**<**.**001**
RBBB, *n* (%)	4 (5.00)	4 (3.39)	0.844

LBBB, left bundle-branch block; LVEF, left ventricular ejection fraction; LVESD, left ventricular internal dimension in systole; LVEDD, left ventricular internal dimension in diastole. The bold values indicate that *P* is less than 0.05, which is considered statistically significant.

Laboratory tests showed that non-recovered patients had higher triglycerides (*P* = 0.017), ApoB (*P* = 0.035), fasting glucose (*P* = 0.036), and NT-proBNP (*P* = 0.001), and lower residual cholesterol (*P* = 0.037) and serum potassium (*P* = 0.001); other indices were comparable. All patients received guideline-directed medical therapy, with more frequent use of SGLT2 inhibitors in the recovered group (72.9% vs. 50%; *P* = 0.001).

Echocardiographic data showed lower baseline LVEF in non-recovered patients [25.8% [IQR: 21.0–30.0] vs. 28.0% [IQR: 25.0–31.0]; *P* = 0.008] and larger LVEDD (7.02 ± 0.87 cm vs. 6.72 ± 0.71 cm); other chamber dimensions were similar. Electrocardiographic findings revealed longer QRS [136 (117.5–160) ms vs. 102 [92–110] ms] and QTc intervals [479 (458.8–511.3) ms vs. 447 (422.0–475.0) ms], and a higher prevalence of LBBB (73% vs. 3%; *P* < 0.001) in non-recovered patients, whereas mean ventricular rate was similar (*P* = 0.678).

#### Predictors of LVEF recovery

Univariable logistic regression analysis identified several factors associated with LVEF recovery from <35% to ≥35% (Table 3).

**Table 3:** Univariate and multivariate firth logistic analysis.

Variables	Univariate		Multivariate Firth	
	OR (95%CI)	*P*	OR (95%CI)	*P*
Gender	1.38 (0.73∼2.61)	0.326		
SGLT-2i	2.69 (1.48∼4.88)	**0**.**001**		
LBBB	0.01 (0.00∼0.04)	**<**.**001**	0.02 (0.00∼0.13)	**<**.**001**
BMI	1.05 (0.98∼1.11)	0.150		
Age	0.95 (0.93∼0.97)	**<**.**001**		
LVEF	1.07 (1.01∼1.13)	**0**.**014**		
RV	1.62 (1.10∼2.39)	**0**.**014**		
LVEDD	0.62 (0.42∼0.90)	**0**.**012**		
TG	1.84 (1.09∼3.11)	**0**.**023**		
TC	1.20 (0.91∼1.59)	0.195		
ApoA1	1.11 (0.98∼1.26)	0.089	1.40 (1.06∼1.86)	**0**.**019**
ApoB	3.35 (1.07∼10.43)	**0**.**037**		
Potassium	3.10 (1.49∼6.45)	**0**.**002**		
Glucose	0.90 (0.77∼1.04)	0.159		
NT-proBNP	0.99 (0.99∼0.99)	**0**.**002**		
QRS time	0.93 (0.91∼0.95)	**<**.**001**		
QTc time	0.98 (0.98∼0.99)	**<**.**001**		

OR, Odds Ratio; CI, Confidence Interval. The bold values indicate that *P* is less than 0.05, which is considered statistically significant.

including SGLT-2 inhibitor use, absence of LBBB, younger age, higher baseline LVEF, larger RV diameter, smaller LVEDD, higher triglycerides (TG) and ApoB, higher fasting serum potassium, lower NT-proBNP, shorter QRS and QTc intervals (*P* < 0.05 for all).

After multivariable Firth logistic regression adjustment, absence of LBBB and higher ApoA1 levels remained independently associated with LVEF recovery to >35%. The presence of LBBB was strongly associated with a lower likelihood of LVEF recovery, while higher ApoA1 levels were associated with a greater likelihood of recovery.

#### Development and validation of the clinical prediction model

**Figure 2:**
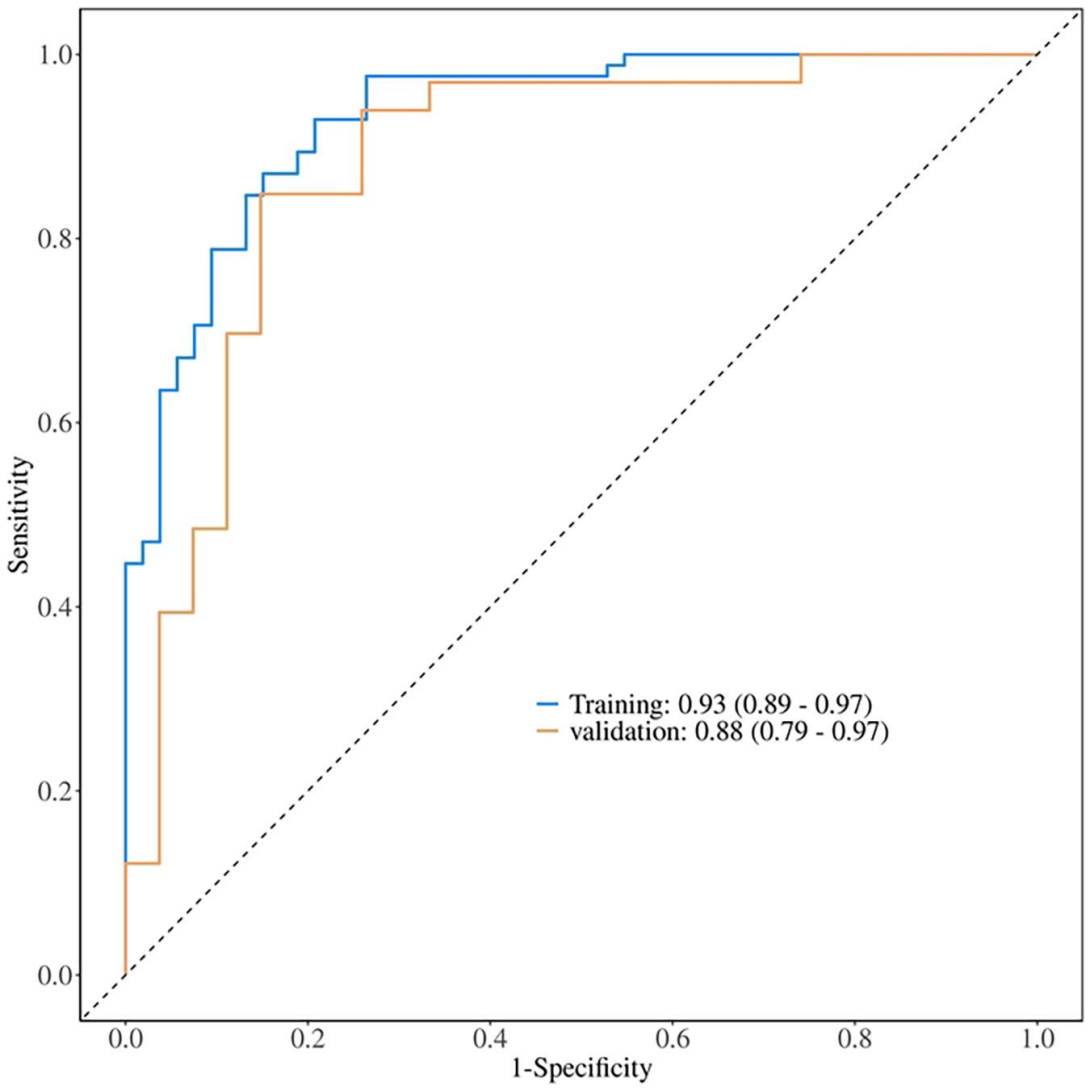
Receiver operating characteristic (ROC) curves of the prediction model in the training and validation sets.

A clinical prediction model was developed using LBBB and ApoA1, with age included as a prespecified clinically relevant variable. The dataset was split into a 7:3 training-validation ratio for model development and internal validation. A logistic regression model was built in the training set using selected predictors. Model performance was evaluated for discrimination (AUC) and calibration (calibration plots and Hosmer–Lemeshow test). The model showed excellent discrimination, with an AUC of 0.93 (95% CI: 0.89–0.97) in the training set and 0.88 (95% CI: 0.79–0.97) in the validation set (Figure 2).

Calibration plots showed good agreement between predicted probabilities and actual outcomes, with Hosmer–Lemeshow test results indicating no significant lack of fit in either the training or validation set (*P* > 0.05). In addition, decision curve analysis (DCA) revealed that the model provided higher net clinical benefit across a range of risk thresholds when compared with “treat-all” and “treat-none” strategies, further supporting its potential utility in clinical decision-making (Supplementary Figure S1).

#### Outcome and survival analysis

**Figure 3:**
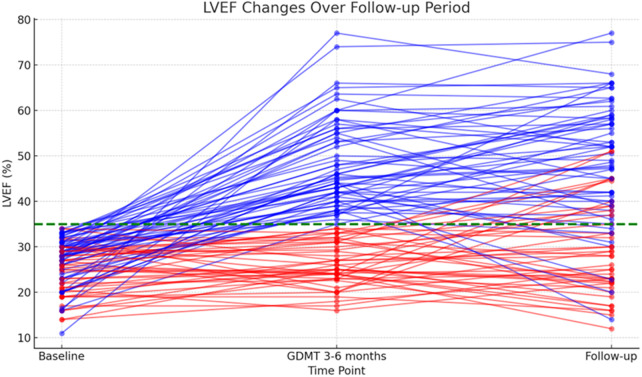
LVEF changes over follow-up period.

Of the 198 enrolled patients, 195 (98.5%) completed telephone follow-up with a median duration of 22.11 months. Ninety-eight patients had at least one echocardiographic re-evaluation. In the non-recovered group (*n* = 42), 10 patients showed LVEF recovery to >35%. In the recovered group (*n* = 56), 16 patients had a decline in LVEF, including 6 whose LVEF fell below 35%, suggesting the need for further ICD evaluation (Figure 3).

Ten patients in the non-recovered group eventually received ICD implantation, and no secondary outcome event was recorded after ICD implantation during follow-up. In the improvement group, one patient underwent prophylactic ICD implantation due to transient syncope and electrocardiographic evidence of LBBB, to prevent SCD. During follow-up, 14 patients in the non-recovered group died, and none had received ICD implantation before death. Of these, 9 deaths (11.3%) occurred within the first 2 years (minimum survival: 4.83 months), and 5 deaths (6.2%) occurred after 2 years. A total of 78 patients in the cohort experienced composite endpoint events.

To explore the association between different clinical characteristics and the occurrence of composite endpoints, univariable Cox regression analysis was first performed (Table 4).

**Table 4:** Univariable and multivariable Cox regression analysis.

Variables	Multivariate		MultivariateMultivariate	
	HR (95%CI)	*P*	HR (95%CI)	*P*
Gender				
Female	1.00 (Reference)			
Male	0.94 (0.58∼1.53)	0.814		
CAD	1.36 (0.76∼2.45)	0.298		
Hypertension	1.12 (0.71∼1.77)	0.624		
Diabetese	0.69 (0.39∼1.20)	0.191		
LBBB	2.20 (1.40∼3.45)	**<**.**001**	3.11 (1.39∼6.99)	**0**.**006**
BMI	0.97 (0.92∼1.02)	0.172		
Age	1.02 (1.01∼1.04)	**0**.**003**	1.02 (1.01∼1.04)	**0**.**004**
LVEF	0.96 (0.92∼0.99)	**0**.**047**		
LV	1.28 (0.93∼1.76)	0.136		
TC	0.81 (0.66∼0.99)	**0**.**048**		
ApoA1	0.88 (0.80∼0.97)	**0**.**009**	0.87 (0.79∼0.95)	**0**.**003**
Potassium	0.98 (0.51∼1.87)	0.946		
NT-proBNP	1.66 (1.06∼2.60)	**0**.**028**		
QRS-time	1.01 (1.01∼1.02)	**0**.**034**	0.99 (0.98∼1.00)	0.150

HR, Hazards Ratio; CI, Confidence Interval. The bold values indicate that *P* is less than 0.05, which is considered statistically significant.

The results showed that LBBB (HR = 2.20, 95% CI: 1.40–3.45, *P* < 0.001), older age (HR = 1.02, 95% CI: 1.01–1.04, *P* = 0.003), lower LVEF (HR = 0.96, 95% CI: 0.92–0.99, *P* = 0.047), lower total cholesterol (HR = 0.81, 95% CI: 0.66–0.99, *P* = 0.048), lower ApoA1 (HR = 0.88, 95% CI: 0.80–0.97, *P* = 0.009), elevated NT-proBNP (HR = 1.66, 95% CI: 1.06–2.60, *P* = 0.028), and prolonged QRS duration (HR = 1.01, 95% CI: 1.01–1.02, *P* = 0.034) were significantly associated with the composite outcome.

After multivariable adjustment, LBBB (HR = 3.11, 95% CI: 1.39–6.99, *P* = 0.006), older age (HR = 1.02, 95% CI: 1.01–1.04, *P* = 0.004), and lower ApoA1 (HR = 0.87, 95% CI: 0.79–0.95, *P* = 0.003) remained independent predictors of the composite endpoint.

**Figure 4:**
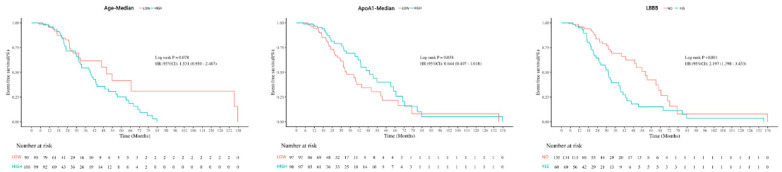
Kaplan–meier curves for event-free survival stratified by Age (high vs. low, based on median split), ApoA1 levels (high vs. low, based on median split), LBBB status.

Kaplan–Meier survival analyses stratified by LBBB status, ApoA1 levels (high vs. low), and age (above vs. below median) showed that patients with LBBB had significantly worse survival (log-rank *P* < 0.001) (Figure 4).

Survival was worse in the low ApoA1 group (*P* = 0.058) and older age group (*P* = 0.078), but the differences did not reach statistical significance. These findings support the adverse prognostic impact of LBBB, while the effects of ApoA1 and age on long-term outcomes need validation in larger cohorts or with extended follow-up.

To enhance individualized prediction of composite endpoints, a Cox proportional hazards model was built using LBBB, age, and ApoA1 levels as independent predictors. Model performance was evaluated for discrimination and calibration at 1-, 3-, and 5-year follow-ups.

**Figure 5:**
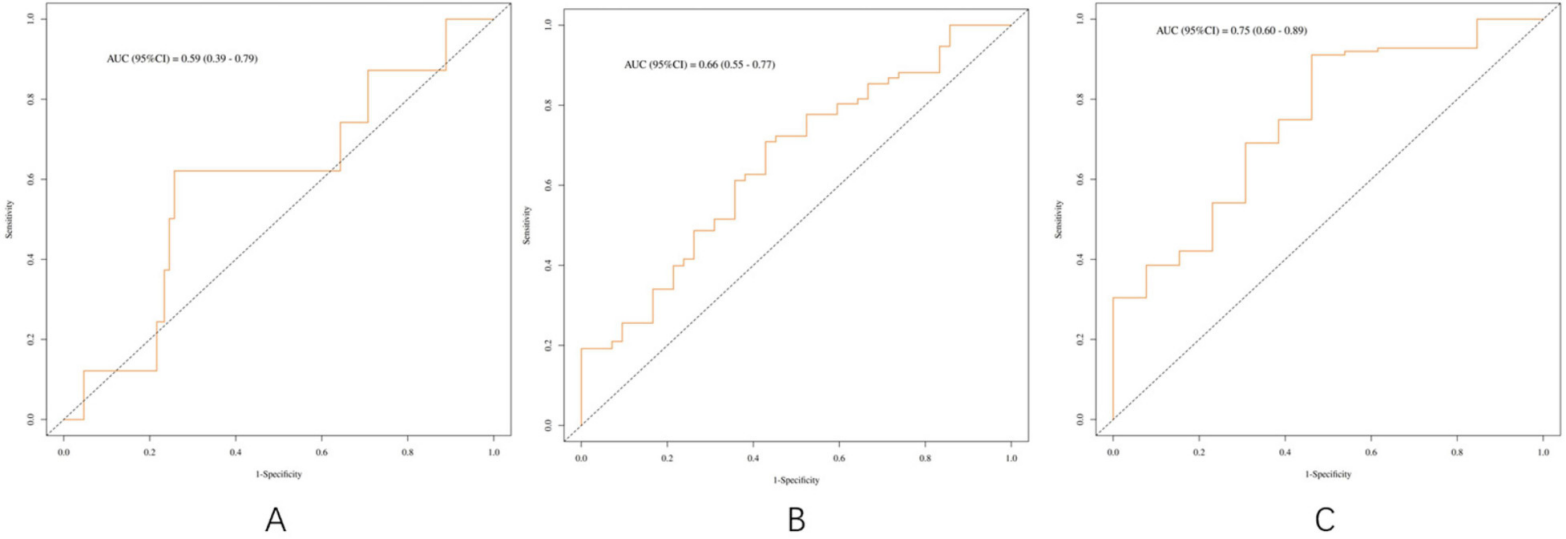
Time-dependent ROC curves of the Cox prediction model at different time points. **(A)** 1-year follow-up;**(B)** 3-year follow-up;**(C)** 5-year follow-up.

Discrimination was assessed with time-dependent ROC curves (Figures 5A–C).

The model yielded area under the curve (AUC) values of 0.59 (95% CI: 0.39–0.79) at 1 year, 0.66 (95% CI: 0.55–0.77) at 3 years, and 0.75 (95% CI: 0.60–0.89) at 5 years, demonstrating a gradual improvement in discriminatory ability over time, with acceptable performance at 5 years.

Calibration was validated via 1,000 bootstrap resamples and assessed using calibration plots. At 1 year, predicted probabilities closely matched observed outcomes, with slight divergence at 3 years in intermediate risk ranges, and strong alignment at 5 years, suggesting robust long-term calibration (Supplementary Figure S2).

The Cox model showed limited short-term discrimination, with a 1-year AUC of 0.59, suggesting that it should not be used for short-term clinical decision-making. Its discriminatory performance improved at 3 and 5 years, indicating that the model may have greater potential value for medium- to long-term risk stratification. These findings should not be interpreted as direct evidence supporting earlier ICD implantation. Rather, the proposed model may support early risk stratification, closer follow-up, repeated LVEF assessment, and timely reassessment of guideline-based device therapy eligibility.

A nomogram was constructed based on three independent predictors—LBBB, age, and ApoA1 levels—to predict the risk of composite endpoint events at 1, 3, and 5 years (Supplementary Figure S3). Higher total points, indicating worse prognosis, were assigned to patients with LBBB, older age, and lower ApoA1 levels, while absence of LBBB, younger age, and higher ApoA1 levels were associated with lower risk and better outcomes. This nomogram provides a practical tool for risk stratification, supporting long-term prognostic assessment and treatment decisions in DCM patients.

## Discussion

This study enrolled 198 treatment-naïve patients with newly diagnosed DCM and a baseline LVEF ≤35% before receiving GDMT, and systematically analyzed clinical characteristics associated with LVEF recovery and adverse prognosis. The results showed that the presence of LBBB was significantly associated with limited LVEF recovery. In multivariable Cox regression analysis, age and ApoA1 levels were important predictors of the composite endpoint, with older age indicating an increased risk of adverse outcomes, whereas lower ApoA1 levels were closely associated with poorer prognosis.

Further analysis suggested that LBBB was not only associated with impaired recovery of cardiac function, but also served as an important risk marker for long-term adverse outcomes. In contrast, although ApoA1 and age showed certain trends in survival analysis, their impact on long-term outcomes requires further validation in larger cohorts with longer follow-up. Overall, this study suggests that age, conduction abnormalities, and metabolism-related indicators may jointly contribute to the recovery of cardiac function and prognostic evolution in patients with DCM, highlighting their potential value for early identification of high-risk patients.

Age was one of the important factors associated with the composite endpoint in this study. With increasing age, the number of cardiomyocytes gradually decreases, regenerative capacity declines, interstitial collagen deposition increases, and myocardial compliance and contractile reserve are progressively reduced (13) In addition, elderly patients often present with chronic low-grade inflammation, abnormal extracellular matrix remodeling, and impaired mitochondrial function, all of which may weaken myocardial reverse remodeling capacity. As a result, older patients may be less likely to achieve significant LVEF improvement after GDMT and may be more prone to adverse events such as heart failure hospitalization and cardiovascular death (14, 15). Therefore, age is not merely a demographic characteristic, but may also reflect myocardial reserve capacity and reparative potential.

The adverse impact of LBBB in patients with DCM may be mainly attributed to electromechanical dyssynchrony and subsequent structural remodeling (16). LBBB can lead to abnormal activation sequences in different segments of the left ventricle, causing some regions to contract prematurely and others to contract with delay. This reduces overall pump efficiency, increases myocardial energy consumption, and aggravates regional mechanical stress. Persistent electromechanical dyssynchrony may further promote ventricular remodeling and myocardial fibrosis, which not only impairs reverse remodeling after GDMT but may also contribute to the development of ventricular arrhythmias (17–19). Therefore, patients with LBBB often exhibit poorer recovery of cardiac function and a higher risk of adverse events (18).

ApoA1 is the major structural protein of high-density lipoprotein and has multiple cardiovascular protective effects, including reverse cholesterol transport, anti-inflammatory and antioxidant actions, and preservation of endothelial function (20). When ApoA1 levels are reduced, the ability to counteract oxidative stress, inflammatory responses, and myocardial fibrosis may be weakened, making the myocardium more vulnerable to persistent injury and thereby promoting ventricular remodeling and deterioration of cardiac function (21). In this study, ApoA1 was independently associated with the composite endpoint, suggesting that it may not only reflect lipid metabolism status but also, to some extent, represent the degree of inflammatory-metabolic imbalance in patients with DCM. Thus, ApoA1 may serve as a potential biomarker for risk stratification and prognostic assessment in this population (22).

In addition, this study observed a higher proportion of SGLT2 inhibitor use in the recovery group, suggesting that optimized pharmacological therapy may contribute to LVEF recovery, which is generally consistent with findings from the DAPA-HF trial (23). SGLT2 inhibitors may exert beneficial effects by improving myocardial energy metabolism, reducing inflammation and fibrosis, promoting natriuresis and diuresis, and facilitating cardiac reverse remodeling (24, 25).

The findings of this study have certain implications for the clinical management of patients with DCM. First, identifying high-risk features such as older age, concomitant LBBB, and lower ApoA1 levels may allow more accurate risk stratification at an early stage of the disease and provide a basis for individualized treatment strategies. For such patients, clinicians should strengthen follow-up monitoring and dynamically assess changes in cardiac function and adverse event risk, rather than relying solely on a single echocardiographic measurement to determine prognosis.

Among patients with LBBB on electrocardiography, pharmacological therapy alone may be insufficient to achieve satisfactory reverse remodeling and LVEF improvement in some cases. In patients with marked conduction abnormalities and limited LVEF recovery, persistent dyssynchronous contraction may cause the left ventricle to continue working inefficiently. Therefore, after adequate optimization of medical therapy, timely reassessment of the need for device therapy, including cardiac resynchronization therapy or implantable cardioverter-defibrillator eligibility, may be warranted to avoid delayed intervention in appropriate high-risk patients.

As an easily accessible laboratory indicator, ApoA1 has promising clinical applicability. If future studies further confirm its stable and reliable predictive value, ApoA1 may be incorporated into comprehensive risk assessment systems for patients with DCM. Combined with clinical characteristics, electrophysiological indicators, and imaging parameters, ApoA1 may help construct more comprehensive prognostic prediction models. Overall, this study suggests that assessment of patients with DCM should not focus solely on LVEF values. Instead, clinical characteristics, conduction abnormalities, metabolic status, and cardiac structural remodeling should be comprehensively considered to more accurately evaluate disease status and guide subsequent treatment.

### Limitation

This study has several limitations. First, its retrospective observational design may have introduced selection bias and residual confounding. Although all patients received GDMT, detailed information on medication titration, target-dose achievement, and long-term adherence was incomplete, and the availability of contemporary four-pillar therapy changed during the study period. Second, DCM is etiologically heterogeneous, but systematic cardiac magnetic resonance imaging, late gadolinium enhancement assessment, and genetic testing were not available for all patients; therefore, DCM etiology, myocardial fibrosis burden, and genetic substrate could not be fully adjusted for. Third, the modest sample size and highly imbalanced distribution of LBBB may have increased the risk of model overfitting. Although internal validation was performed, external validation in larger multicenter cohorts is required. Fourth, the temporal relationship among LVEF reassessment, adverse events, and ICD implantation could not be fully reconstructed; thus, our findings should not be interpreted as direct evidence supporting earlier ICD implantation, but rather as supporting risk stratification, closer follow-up, and timely reassessment of guideline-based ICD eligibility. Fifth, the composite endpoint included heterogeneous events, and the Cox model showed limited short-term discrimination, particularly at 1 year. Finally, ApoA1 may reflect systemic inflammation, nutritional status, frailty, or other unmeasured metabolic factors rather than a direct causal mechanism. Inflammatory markers such as CRP and serum sodium were not consistently available for all patients and were therefore not included in the primary models. Although patients with severe primary valvular heart disease or significant cardiovascular volume overload were excluded, detailed assessment of milder valvular abnormalities was not systematically incorporated into the prediction models. These factors may influence LVEF recovery and clinical outcomes and should be evaluated in future prospective studies. Future prospective studies incorporating standardized serial imaging, detailed GDMT data, cardiac magnetic resonance imaging, genetic testing, dynamic biomarkers, and external validation are warranted.

## Conclusion

In conclusion, LBBB and lower ApoA1 levels were independently associated with failure of LVEF recovery after 3–6 months of GDMT in patients with newly diagnosed DCM and baseline LVEF ≤35%. LBBB, older age, and lower ApoA1 levels were also associated with adverse long-term outcomes. The proposed model may help identify patients at higher risk of persistent LV systolic dysfunction and support closer follow-up, repeated echocardiographic assessment, and timely reassessment of guideline-based device therapy eligibility. Given the retrospective design, modest sample size, lack of external validation, and inability to fully reconstruct the exact temporal relationship among LVEF reassessment, adverse events, and ICD implantation, these findings should be considered exploratory and require confirmation in larger prospective multicenter cohorts.

## Data Availability

The original contributions presented in the study are included in the article/Supplementary Material, further inquiries can be directed to the corresponding author.
