# Supplementary material for: Predictors of left ventricular ejection fraction recovery after guideline-directed medical therapy in patients with newly diagnosed dilated cardiomyopathy and baseline LVEF ≤35%
Source: Front Cardiovasc Med. 2026 Jun 12;13:1767079. doi: 10.3389/fcvm.2026.1767079 (PMC13306976; doi:10.3389/fcvm.2026.1767079)
Supplement: Supplementary file 1 [file Table1.docx]

## Supplementary Table S1 Key R packages and functions used

| Purpose | Package | Principal functions |
| --- | --- | --- |
| Descriptive statistics & basic tests | stats | t.test, wilcox.test, chisq.test, fisher.test, glm |
| Dataset partitioning | caret | createDataPartition |
| Logistic regression model building | stats | glm; stepwise selection with step |
| Discrimination (AUC) | pROC | roc, auc |
| Calibration & bootstrap | rms | calibrate, val.prob, plotting functions |
| Goodness‑of‑fit | ResourceSelection | hoslem.test |
| Decision‑curve analysis | DecisionCurve | decision_curve, plot_decision_curve |
| Survival modelling | survival | coxph, cox.zph, survfit, survdiff |
| Time‑dependent ROC | timeROC | timeROC |
| Reproducibility | base | set.seed(1234) |
