# Supplementary material for: Predictors of left ventricular ejection fraction recovery after guideline-directed medical therapy in patients with newly diagnosed dilated cardiomyopathy and baseline LVEF ≤35%
Source: Front Cardiovasc Med. 2026 Jun 12;13:1767079. doi: 10.3389/fcvm.2026.1767079 (PMC13306976; doi:10.3389/fcvm.2026.1767079)
Supplement: Supplementary file 2 [file Table2.docx]

**Supplementary Table 2. Longitudinal changes in LVEF according to recovery status after 3–6 months of GDMT**

| **Variable** | **Overall,**  **n = 98** | **Non-recovered group,**  **n = 42** | **Recovered group,**  **n = 56** | **P value** |
| --- | --- | --- | --- | --- |
| Baseline LVEF, % | 27.85 (22.00–30.00) | 25.75 (20.63–29.00) | 28.00 (23.75–31.00) | 0.026 |
| LVEF after 3–6 months of GDMT, % | 38.00 (27.00–46.75) | 25.00 (23.00–30.75) | 45.65 (41.00–55.25) | <0.001 |
| Last follow-up LVEF, % | 40.00 (28.00–52.75) | 27.00 (22.08–36.50) | 52.00 (40.90–59.00) | <0.001 |
| ΔLVEF from baseline to 3–6 months, % | 11.00 (2.00–20.75) | 1.00 (-1.23–4.00) | 18.00 (12.88–26.00) | <0.001 |
| ΔLVEF from 3–6 months to last follow-up, % | 1.00 (-4.00–9.00) | 1.50 (-3.00–7.93) | 1.00 (-4.25–10.15) | 0.760 |
| ΔLVEF from baseline to last follow-up, % | 12.25 (2.48–25.00) | 4.00 (-2.78–9.00) | 22.00 (13.15–32.75) | <0.001 |
| Late LVEF decline, n (%) | 42 (42.9) | 18 (42.9) | 24 (42.9) | 1.000 |
| Last follow-up LVEF >35%, n (%) | 59 (60.2) | 11 (26.2) | 48 (85.7) | <0.001 |
| Last follow-up LVEF ≤35%, n (%) | 39 (39.8) | 31 (73.8) | 8 (14.3) | <0.001 |

**note：**Values are presented as median (interquartile range) or n (%). The non-recovered and recovered groups were defined according to LVEF recovery to >35% after 3–6 months of GDMT. ΔLVEF values represent absolute percentage-point changes in LVEF. Late LVEF decline was defined as a lower LVEF at the last follow-up than at the 3–6-month reassessment. P values were calculated using the Mann–Whitney U test for continuous variables and Fisher’s exact test for categorical variables.
