# Supplementary material for: Predictors of left ventricular ejection fraction recovery after guideline-directed medical therapy in patients with newly diagnosed dilated cardiomyopathy and baseline LVEF ≤35%
Source: Front Cardiovasc Med. 2026 Jun 12;13:1767079. doi: 10.3389/fcvm.2026.1767079 (PMC13306976; doi:10.3389/fcvm.2026.1767079)
Supplement: Supplementary file 3 [file presentation1.pptx]

## Slide 1
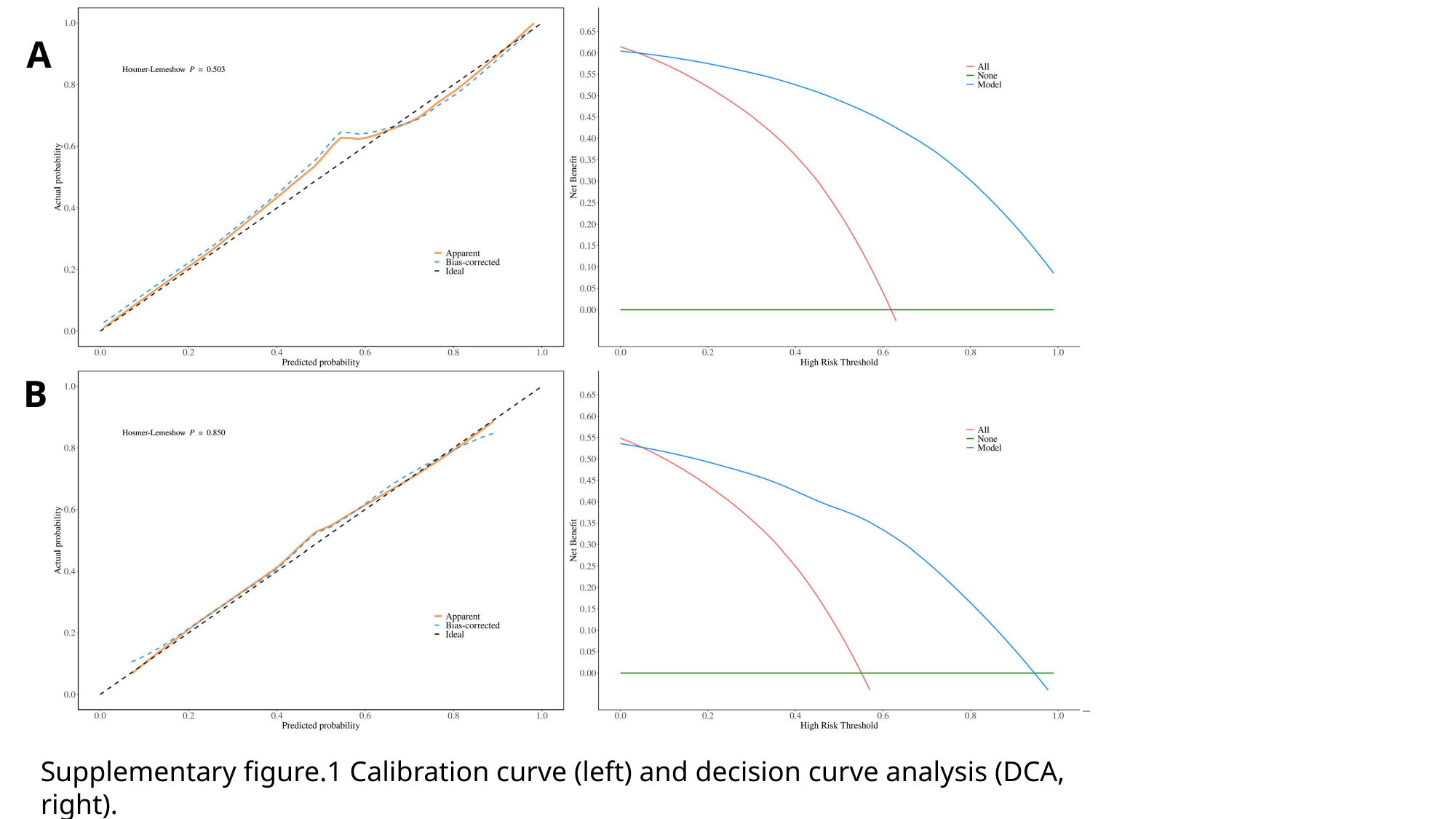

A
B
Supplementary figure.1 Calibration curve (left) and decision curve analysis (DCA, right).
 (A) Training set ;(B)Validation set.

## Slide 2
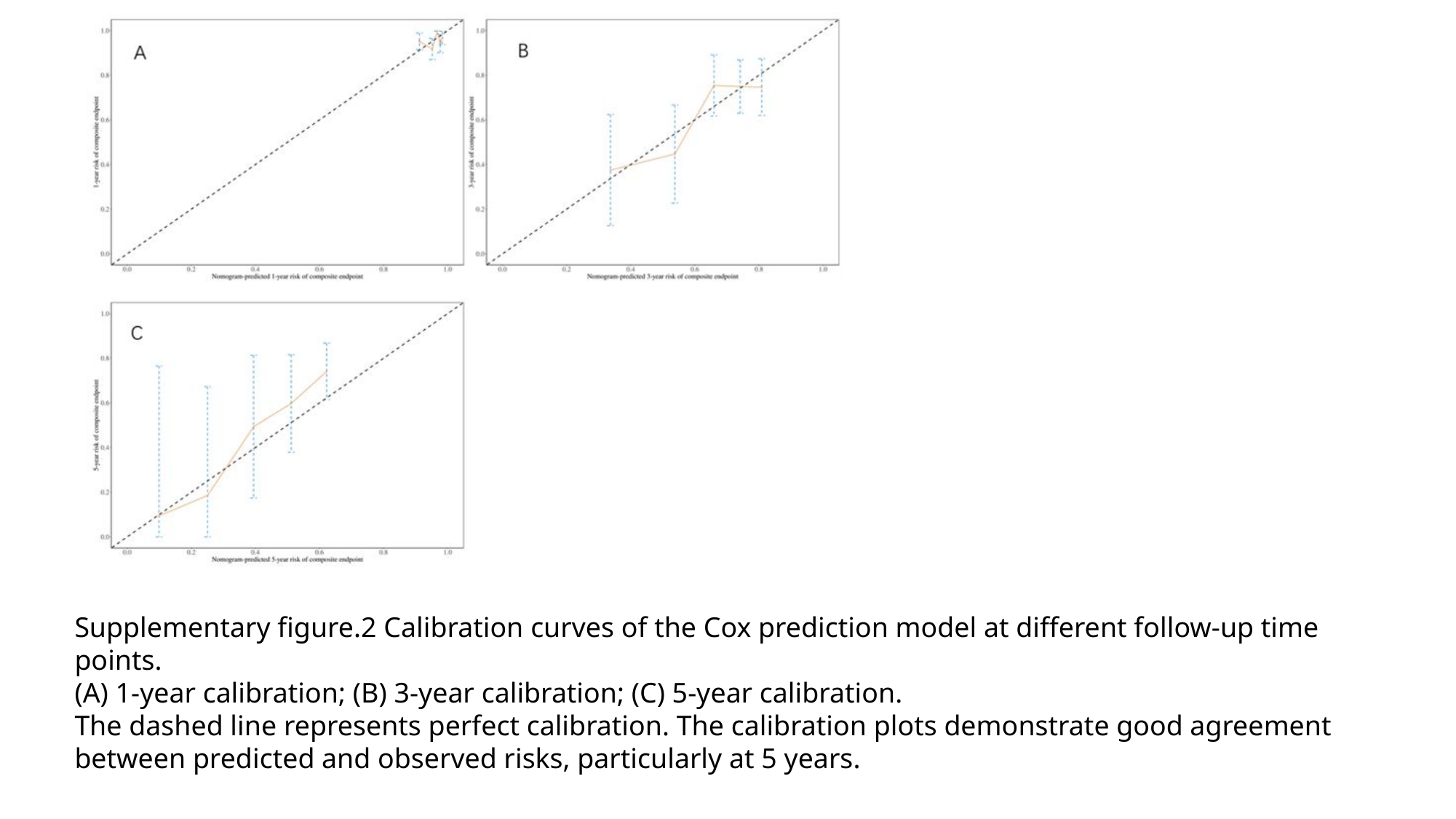

Supplementary figure.2 Calibration curves of the Cox prediction model at different follow-up time points.
(A) 1-year calibration; (B) 3-year calibration; (C) 5-year calibration.
The dashed line represents perfect calibration. The calibration plots demonstrate good agreement between predicted and observed risks, particularly at 5 years.

## Slide 3
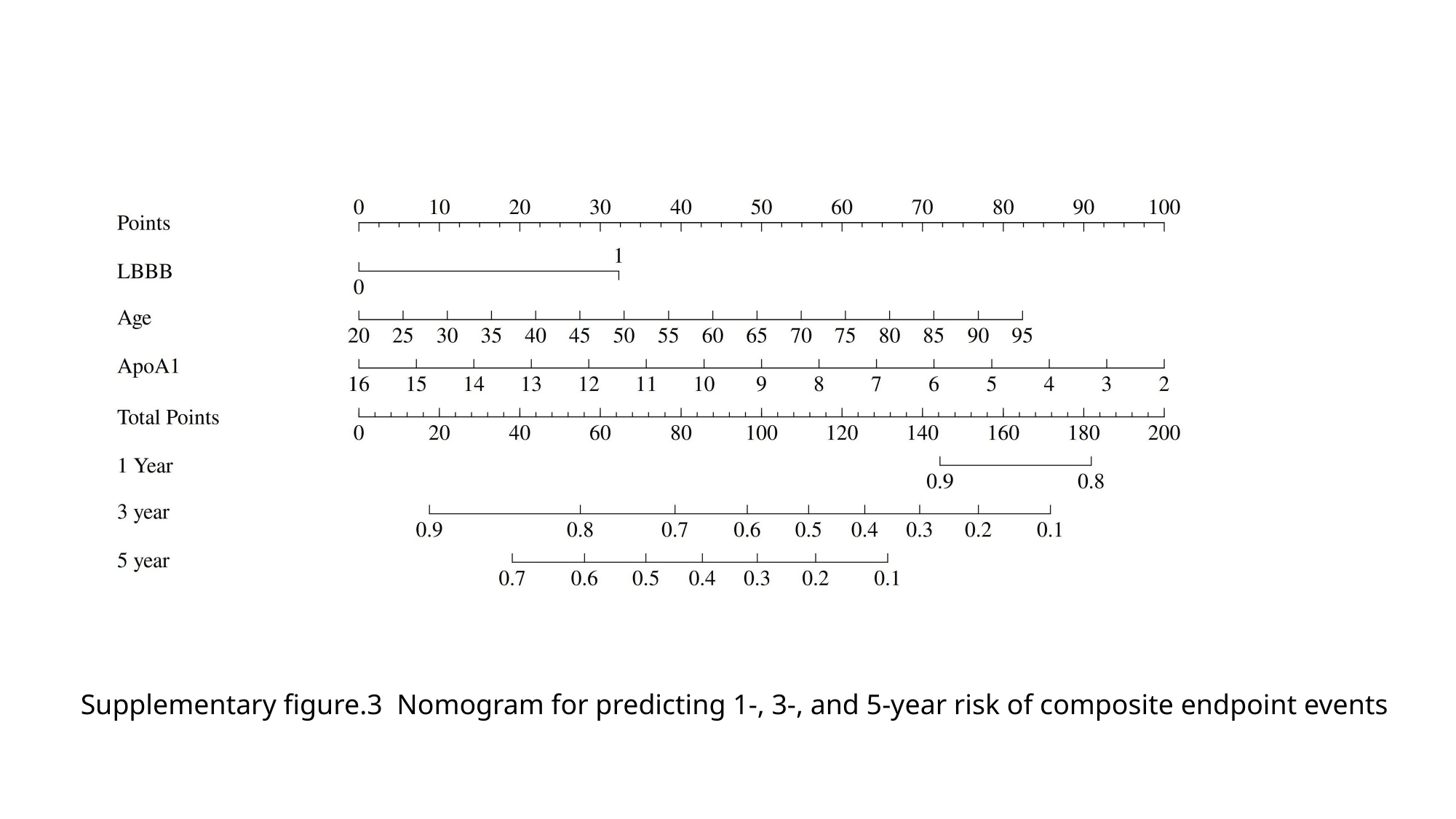

Supplementary figure.3 Nomogram for predicting 1-, 3-, and 5-year risk of composite endpoint events
